# Effect of a Preparation of Four Probiotics on Symptoms of Patients with Irritable Bowel Syndrome: Association with Intestinal Bacterial Overgrowth

**DOI:** 10.1007/s12602-018-9401-3

**Published:** 2018-03-05

**Authors:** Konstantinos Leventogiannis, Paraskevas Gkolfakis, Georgios Spithakis, Aikaterini Tsatali, Aikaterini Pistiki, Athanasios Sioulas, Evangelos J. Giamarellos-Bourboulis, Konstantinos Triantafyllou

**Affiliations:** 1 4th Department of Internal Medicine, National and Kapodistrian University of Athens, Athens, Greece; 2 Hepatogastroenterology Unit, 2nd Department of Internal Medicine-Propedeutic, Research Institute and Diabetes Center, National and Kapodistrian University of Athens, Athens, Greece; 3 4th Department of Internal Medicine, ATTIKON University Hospital, 1 Rimini Street, 1262 Athens, Greece

**Keywords:** Irritable bowel syndrome, Intestinal bacterial overgrowth, Probiotics

## Abstract

The effect of probiotics on small intestinal bacterial overgrowth (SIBO) in irritable bowel syndrome (IBS) has never been studied so far. In this prospective trial, five patients with IBS and SIBO and 21 patients with IBS without SIBO were administered an oral capsule containing *Saccharomyces boulardii*, *Bifidobacterium lactis*, *Lactobacillus acidophilus*, and *Lactobacillus plantarum* (Lactolevure^®^) every 12 h for 30 days. SIBO was defined by quantitative culture of the third part of the duodenum; IBS was defined by the Rome III criteria. Severity of symptoms was graded by the IBS severity scoring system (SSS). The primary study endpoint was the efficacy of probiotics in improvement of symptoms of IBS in patients with SIBO. Thirty days after the end of treatment, a 71.3% decrease of the total IBS score was detected in patients with IBS and SIBO compared to 10.6% in those without SIBO (*p* 0.017). A similar decrease was achieved among patients with constipation-predominant IBS without SIBO. Post-treatment satisfaction from bowel function was greater in patients with SIBO. Similar satisfaction improvement was found among patients with diarrhea-predominant IBS irrespective from SIBO; pain intensity score decreased in patients with constipation-predominant IBS irrespective from SIBO. The benefit of probiotics was greater among patients with a pro-inflammatory cytokine pattern in the duodenal fluid. This is the first study that prospectively demonstrated superior clinical efficacy of probiotics in patients with IBS with SIBO. Analysis also showed considerable benefit from probiotic intake regarding certain symptoms of patients with diarrhea-predominant and constipation-predominant IBS.

Trial registration: ClinicalTrials.gov identifier NCT02204891.

## Introduction

Irritable bowel syndrome (IBS) is the most common functional gastrointestinal disorder. Pathogenesis remains multifactorial. Better understanding of the interaction of the host with intestinal microbiota the last decade led to the knowledge that many of the symptoms of IBS, mainly bloating and diarrhea, are related with the overgrowth of bacteria of colonic type in the small intestine. This overgrowth frames the syndrome of small intestinal bacterial overgrowth (SIBO) where colonic type of bacteria predominates in the proximal parts of the small intestine. Fermentation of dietary carbohydrates by the bacteria colonizers of SIBO ends with the over-production of gas generating thus symptoms of IBS. The relationship between IBS and SIBO was found by a series of prospective observational studies using the lactulose and the glucose breath tests for the diagnosis of SIBO [1–4]. Using these tests, the prevalence of SIBO in patients with IBS ranged between 65 and 85%. We conducted a large-scale study on 897 patients undergoing upper GI tract endoscopy collecting samples from the third part of the duodenum for quantitative culture. The frequency of SIBO ranged between 10.6 and 17.6% depending on the cutoff used for the diagnosis [5]. Our data also showed that among patients with IBS the prevalence of SIBO defined at a cutoff of 10^3^ cfu/ml was 37.5% [6].

Oral supplementation with probiotics may be a candidate approach for the eradication of SIBO and subsequently of the symptoms of IBS. The majority of probiotic bacteria belong to the *Lactobacillus* and *Bifidobacterium* genera. They are Gram-positive lactic acid-producing bacteria that constitute a major part of the normal human intestinal microflora. The rationale behind their use as a therapeutic strategy in IBS is that orally administered probiotics may replace the overgrown enteric-type bacteria of SIBO. Four randomized clinical trials are available evaluating the efficacy of orally administered probiotics in IBS. The common findings of these trials are as follows: (a) they improve the symptoms of bloating and diarrhea which are typical of the presence of SIBO; and (b) efficacy is usually detected when mixtures of different species of probiotics are used [7–10]. However, irrespective of the above results, there is lot of ambiguity on the efficacy of probiotics in IBS because not all conducted studies administered the same probiotic species whereas no evidence is provided on a possible effect of probiotics on the mechanisms of pathogenesis of IBS.

The aim of the present study is to evaluate the effect of a mixture of four species of probiotics (*Saccharomyces boulardii*, *Bifidobacterium lactis* BB-12, *Lactobacillus acidophilus* LA-5, and *Lactobacillus plantarum*) on the symptoms of IBS patients with culture verified SIBO compared to those without SIBO. The selection of these probiotic species was based on the fact that they are well-described commensals of the proximal intestinal flora even though normal bacterial microbiota is more stable than the mycobiota [11, 12]. Prebiotics were not studied since many of patients with IBS present with fructose intolerance whereas diets low in oligo-saccharides have been shown beneficiary for these patients [13]. We anticipated that findings might provide an insight on the role of probiotics in treating one of the mechanisms of pathogenesis of IBS.

## Patients and Methods

### Study Design

This was an open-label clinical study that was conducted during the period April 2014–September 2016 in patients followed in the Hepatogastroenterology Unit of the 2nd Department of Internal Medicine-Propedeutic and the Outpatients Department of Immunology of Infections of the 4th Department of Internal Medicine of ATTIKON University Hospital in Greece. The study protocol was approved by the Institutional Review Board of the Hospital (approval 4/19-03-2014) and it was registered at www.clinicaltrials.govbefore enrolment of the first patient (ClinicalTrials.gov identifier NCT02204891). All patients were screened after written informed consent.

Inclusion criteria were as follows: (a) age ≥ 18 years; (b) patients of both genders; (c) written informed consent by study participants; and (d) presence of IBS according to Rome III criteria [14]. Exclusion criteria were as follows: (a) age below 18 years; (b) deny to consent; (c) pregnancy or lactation; (d) history of inflammatory bowel disease; (e) any GI tract infection during the last 2 months; (f) diabetes mellitus type 1 or type 2; (g) use of laxatives and antibiotics within the preceding 6 weeks; (h) presence of fever, abdominal mass, signs of bowel obstruction, and/or leucocytosis; (i) abnormal serum levels of thyroid-stimulating hormone (TSH); (j) history of colon cancer or colon diverticula; (k) infection by the human immunodeficiency virus (HIV), hepatitis B virus (HBV), and hepatitis C virus (HCV); (i) celiac disease identified by biopsy of the duodenal mucosa; (m) history of scleroderma and gastroparesis; (n) planning pregnancy within the next 3 months.

Five milliliters of blood was drawn after venipuncture of one forearm vein under aseptic conditions for the measurement of TSH and for serology for HIV, HBV, and HCV from all IBS patients meeting all the inclusion and exclusion criteria. If TSH was within the normal range and serology was negative, the patient underwent colonoscopy. After negative for cancer and diverticula colonoscopy, the patient underwent upper GI tract endoscopy. During endoscopy, upon entrance of the endoscope at the third section of duodenum, intestinal fluid was aspirated. Aspirate was collected by placing a sterile suction catheter inside a sterile overtube, which was passed through the suction channel of the endoscope. The aspirate was placed immediately in transport vials and transported to the laboratory for quantitative cultures. At the same time, three duodenal biopsy samples were obtained to exclude celiac disease.

Cultures of duodenal fluid were done, as reported elsewhere [5, 6]. When the results of quantitative duodenal culture were available, patients could be divided into those with SIBO and into those without SIBO. For the needs of the study, SIBO was defined as any equal to or more than 10^5^ CFU/ml of duodenal aspirate and/or presence of colonic type bacteria in the duodenal aspirate. Concentrations of tumor necrosis factor-alpha (TNFα), interleukin (IL)-6 and IL-8 were measured in the duodenal fluid by an enzyme immunosorbent assay (R&D Inc., Minneapolis MN); the lower detection limit was 3.9 pg/ml for all cytokines.

### Interventions and Study Visits

When a patient was enrolled in the study, study procedures and follow-up visits were done by study physicians who were completely blind to the information if the patient had SIBO or not. At enrolment, patients were classified based on their symptoms into diarrhea-predominant IBS (IBS-D), constipation-predominant IBS (IBS-C), and mixed-type IBS (IBS-M). They were also asked to report the total number of days on which they felt abdominal pain the last 15 days. Then patients were subject to three visits. At visit 1, patients were provided with 60 capsules of a commercially available preparation of four probiotics containing per capsule 1.5 × 10^9^ cfu *Saccharomyces boulardii*, 1.75 × 10^9^ cfu *Bifidobacterium lactis* BB-12, 1.5 × 10^9^ cfu *Lactobacillus acidophilus LA-5*, and 0.5 × 10^9^ cfu *Lactobacillus plantarum* (Lactolevure^®^, Uni-Pharma S.A., Athens, Greece). Capsules were administered orally every 12 h 30 min before meal for 30 days. At the end of treatment, patients were subject to visit 2 on day 30 ± 7 where they had to provide the empty capsule cartridges as a proof of compliance with treatment. Patients were also coming for visit 3 on day 60 ± 7 for follow-up. On visit 1 before start of treatment, on visit 2, and on visit 3, patients were asked to complete a modified IBS Severity Scoring System (IBS-SSS) questionnaire in the Greek language that was composed of five questions on the current intensity of abdominal pain, the overall intensity of abdominal pain, the intensity of bloating, the effect of IBS on the quality of life, and on the level of satisfaction from the bowel function [15]. Each question was self-assessed from 0 to 100 so that the IBS-SSS was ranging from 0 to 500. For the question of the effect of IBS on the quality of life, it was explained to the patients that 0 represented no effect and 100 represented complete effect. For the question of the level of satisfaction from the bowel function, it was explained to the patients that 0 represented full satisfaction and 100 the complete lack of satisfaction. On each visit, patients were demonstrated a chart with the Bristol stool scale [16] and they were asked to score the form of their stool the last 7 days at a scale from 1 to 7. The days from work abstinence due to IBS were also reported. Occurrence of adverse events was recorded over study visits.

### Study Endpoints

The primary study endpoint was the rate of patients with IBS and SIBO achieving any ≥ 50% decrease of IBS-SSS compared to patients with IBS without SIBO on visit 3. This cutoff of decrease of IBS-SSS was selected according to published criteria of significant efficacy [15]. Four secondary endpoints were set: (a) changes of the IBS-SSS among patients without SIBO; (b) the comparisons on the five components of the IBS-SSS; (c) the effect on the form of stool as assessed by the Bristol stool scale; and (d) the effect on the number of lost days of work. The association between the level of cytokines in the duodenal fluid and the change of IBS-SSS was an exploratory endpoint. All four secondary endpoints were compared among patients with the three IBS subtypes and on both study visits.

### Power of the Study

The study was powered for the primary endpoint assuming that (a) symptoms of IBS would be improved in 20% of SIBO-negative patients; and (b) SIBO would be eradicated in 80% of patients. To demonstrate this difference 10% significance with 80% power, it was calculated that 30 patients with IBS and SIBO and that 30 patients with IBS without SIBO should be enrolled. However, awareness of the extensive exclusion criteria and of the strict definition of SIBO using cutoff 10^5^ cfu/ml that could limit the enrolment rate, a first read-out for the primary endpoint was done in December 2015 to decide on the progress of the study.

### Statistical Analysis

Frequencies were reported as percent and 95% confidence interval (CI). Comparisons between groups of patients with ≥ 50% decrease of the IBS-SSS were done by the Fisher exact test. Quantitative variables were reported as mean ± SD. Percent changes of quantitative variables from visit 1 were calculated and reported as means ± SE. Comparisons were done by the Mann-Whitney *U* test. Percentiles of the concentrations of TNFα were determined. Patients with TNFα in the duodenal fluid above the 75% percentile or with all three measured cytokines above the lower limit of detection were considered to be pro-inflammatory; remaining patients were considered normal. Comparison of the achievement of 50% decrease of the IBS-SSS between pro-inflammatory and normal patients was done by the binomial test. Any value of *p* below 0.05 was considered statistically significant.

## Results

**Fig. 1:**
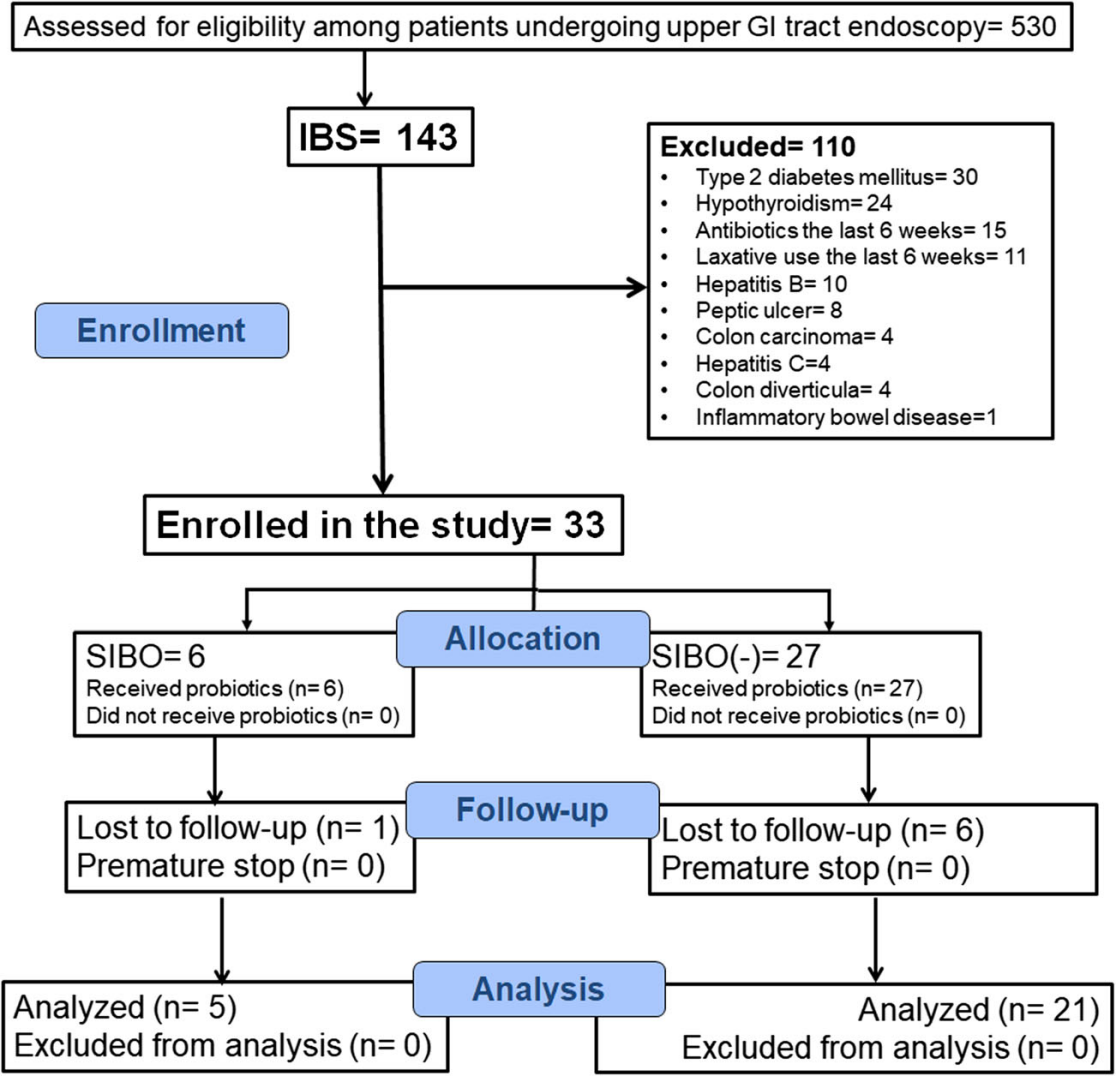
Study CONSORT flowchart. Abbreviations: *GI*, gastrointestinal; *IBS*, irritable bowel syndrome; *SIBO*, syndrome of intestinal bacterial overgrowth

The enrolment rate for the study was low due to the strict enrolling criteria allowing enrolment of less than one out of four screened patients (Fig. 1). Since analysis for the primary efficacy endpoint conducted in December 2015 showed a trend towards statistical difference between patients with SIBO and patients without SIBO, it was allowed for the study to enroll patients for another 6 months. A total of 33 patients were enrolled, 6 with SIBO and 27 without SIBO. Five and 21 patients respectively attended all study visits and could be analyzed. Among the five patients with SIBO, four patients had growth of *Escherichia coli* at counts exceeding 10^7^ cfu/ml and one had growth of *Klesbsiella pneumoniae* at counts exceeding 10^7^ cfu/ml (Table 1).

**Table 1:** Baseline demographics of enrolled patients

	SIBO(−) (*n* = 21)	SIBO(+) (*n* = 5)	*p* value
Male/female	4/17	1/4	1.000
Age (years, mean ± SD)	50.8 ± 15.1	53.8 ± 9.9	0.696
Number of days with abdominal pain the last 15 days (mean ± SD)	5.67 ± 3.23	5.00 ± 3.81	0.691
Type of IBS (*n*, %)			
Diarrhea-predominant	5 (23.8)	4 (80.0)	
Constipation-predominant	7 (33.3)	0 (0)	0.148
Mixed-type	9 (42.9)	1 (20.0)	
IBS-SSS (mean ± SD)	202.8 ± 113.4	208.0 ± 78.5	0.925
Components of IBS-SSS (mean ± SD)			
Current abdominal pain intensity	22.0 ± 35.0	24.0 ± 16.9	0.506
Abdominal pain intensity	20.5 ± 31.0	22.0 ± 19.2	0.919
Bloating intensity	30.0 ± 28.1	20.0 ± 20.0	0.464
Satisfaction from bowel function	53.0 ± 31.6	40.0 ± 28.8	0.700
Effect on daily life	66.5 ± 28.1	80.0 ± 12.2	0.311
Bristol stool form scale (mean ± SD)	3.45 ± 1.98	3.40 ± 2.40	0.962
Days of work abstinence/week due to IBS (median, range)	0.5 (0–52)	0.5 (0–32)	1.000
TNFα in the duodenal fluid (pg/ml, mean ± SE)	45.4 ± 6.2	51.9 ± 13.8	0.696
IL-6 in the duodenal fluid (pg/ml, mean ± SE)	6.2 ± 2.3	6.5 ± 3.1	0.629
IL-8 in the duodenal fluid (pg/ml, mean ± SE)	27.8 ± 20.2	31.9 ± 22.3	0.959

*IBS-SSS* irritable bowel syndrome severity scoring system, *SIBO* small intestinal bacterial overgrowth

**Fig. 2:**
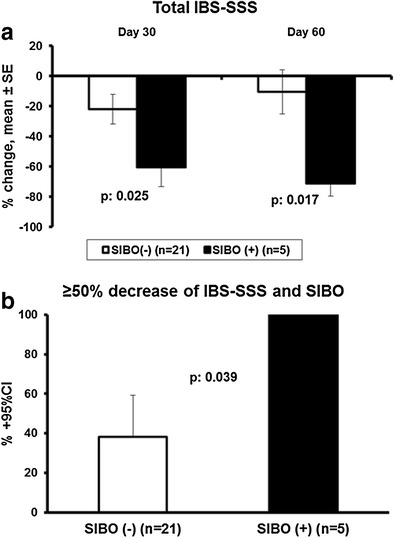
Study primary endpoint: effect of treatment with the studied preparation of four probiotics on the total irritable bowel syndrome severity scoring system (IBS-SSS) in relation to the presence of small intestinal bacterial overgrowth (SIBO). **a** Percent change of baseline total IBS-SSS at the end of treatment (30 days) and on follow-up (60 days). *p* values of comparisons between patients without and with SIBO are provided. **b** Comparison of the rate of patients with at least 50% decrease of total IBS-SSS on follow-up visit between patients without and with SIBO. The *p* value of comparison is shown. *CI*, confidence interval

The study was successful in the primary endpoint showing mean 71.3% decrease of the total IBS-SSS on visit 3 among patients with IBS and SIBO compared to 10.6% among patients with IBS without SIBO (*p* 0.017) (Fig. 2a). All patients with SIBO presented with ≥ 50% decrease of the total IBS-SSS on visit 3 compared to 38.1% of patients without SIBO (*p* 0.039) (Fig. 2b).

**Fig. 3:**
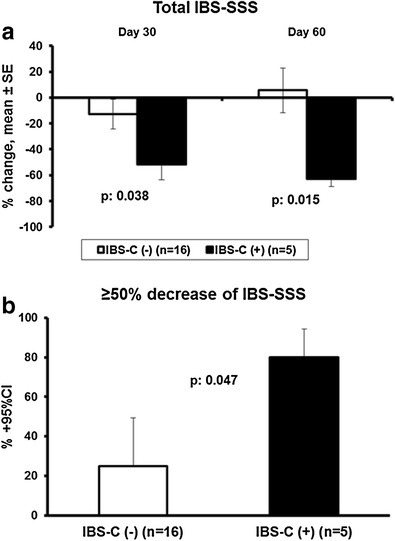
Effect of treatment with the studied preparation of four probiotics on patients with IBS without small intestinal bacterial overgrowth**. a** Percent change of baseline total IBS score at the end of treatment (30 days) and on follow-up (60 days). **b** Comparison of the rate of patients with at least 50% decrease of total IBS SS on follow-up visit between patients without and with predominant constipation IBS-C. The *p* value of comparison is shown. *CI*, confidence interval

Analysis of the first secondary endpoint that included patients without SIBO revealed considerable benefit from probiotic intake among patients with IBS-C. More precisely, mean 62.7% decrease of the total IBS-SSS on visit 3 among patients with IBS-C was shown compared to 5.6% increase among patients with IBS without constipation (*p* 0.015) (Fig. 3a). Eighty percent of patients with IBS-C presented with ≥ 50% decrease of the total IBS-SSS on visit 3 compared to 25% of patients without SIBO (*p* 0.047) (Fig. 3b).

**Fig. 4:**
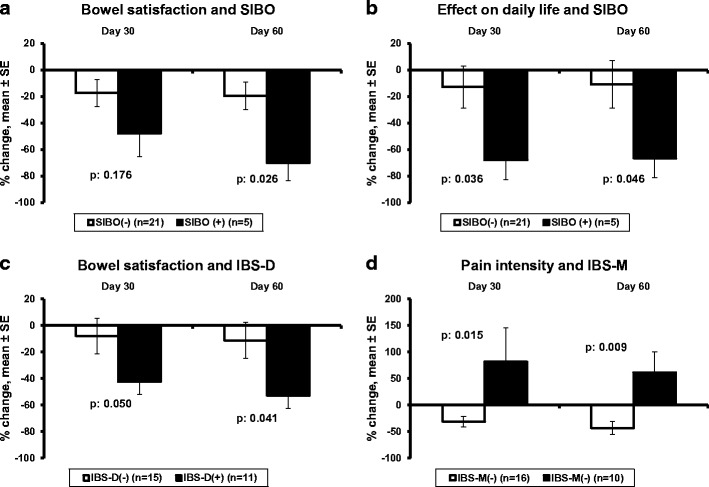
Effect of treatment with the studied preparation of four probiotics on the components of the IBS Severity Scoring System. **a**, **b** Percent change of scoring for bowel satisfaction and for the effect of bowel function on daily life in relation to the absence or presence of small intestinal bacterial overgrowth (SIBO). **c** Percent change of scoring for bowel function satisfaction in relation to the presence of diarrhea predominant IBS (IBD-D). **d** Percent change of abdominal pain intensity in relation to the presence of mixed-type IBS (IBD-M) The *p* values of statistically significant comparisons are shown

The second secondary endpoint was the comparisons of the over-visits changes of each of the quantitative components of the IBS-SSS. Figure 4 shows only significant changes between subgroups of patients. The scores for bowel function satisfaction and for the effect of IBS on the quality of life were significantly decreased among patients with SIBO (Fig. 4a, b). As already stated, for both these components of the IBS-SSS, the greater score signifies less satisfaction and more effect on the quality of life respectively so that the effected decreases indicate improvement. The score for bowel function satisfaction was also decreased among patients with IBS-D (Fig. 4c). Decrease of the abdominal pain intensity score was detected among patients without IBS-M (Fig. 4d).

**Fig. 5:**
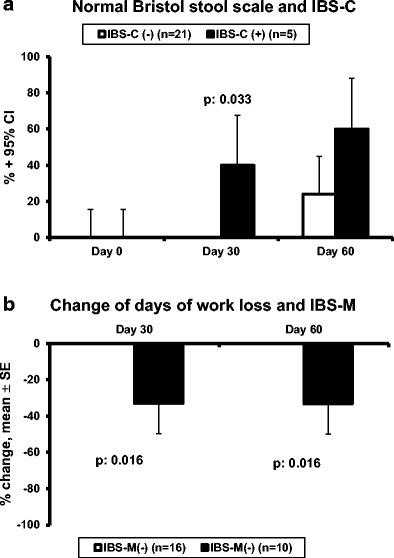
Effect of treatment with the studied preparation of four probiotics on the stool form and on the days of work loss. **a** Rate of patients with normal stool in relation to the presence of constipation-predominant IBS (IBS-C). Normal stool is considered as any Bristol stool scale of 4 or 5. **b** Decrease of the days of work loss compared to the period before treatment in relation to the presence of mixed-type IBS (IBS-M). The *p* values of statistically significant comparisons are shown. *CI*. confidence interval

The third secondary endpoint was the effect of probiotic treatment on the form of stool as assessed by the Bristol stool scale. Any scale of 4 and 5 is considered normal defecation [16, 17]. According to this, the rate of patients achieving normal defecation was greater among patients with IBS-C (Fig. 5a). A salient benefit from probiotic intake was found among patients with IBS-M regarding the fourth secondary study endpoint. Among these patients, the days of work loss were significantly decreased (Fig. 5b).

Ten patients were pro-inflammatory based on their cytokine patterns in the duodenal fluid and 16 were normal; any ≥ 50% decrease of the IBS-SSS in visit 3 was achieved in seven (70%) and six (37.5%), respectively (*p* 0.007).

No adverse events were reported.

## Discussion

Our study differs considerably regarding its design compared to the published trials that aimed to detect efficacy of probiotic preparations on the improvement of symptoms of IBS. The vast majority of conducted clinical trials with probiotics are based on the assumption that the efficacy of probiotics may rely on the substitution of colonic-type bacteria colonizing the upper GI tract of the patients that cause SIBO with the administered probiotics. In these studies, the improvement of bloating is used as indirect evidence for the modulation of SIBO, since bloating is a major symptom of SIBO. However, no direct evidence for an effect of probiotics on SIBO has been published yet. The current non-randomized study aimed to provide direct evidence of the effect of probiotics on SIBO among patients with IBS. Our results showed a statistically greater reduction of the IBS-SSS score among IBS patients with SIBO than among those without SIBO. Moreover, subgroup analysis revealed IBS-C and IBS-D patients to be those the most benefitted target populations for probiotics treatment; patients with IBS-D and IBS-C presented post-treatment with great satisfaction for their bowel function scores and with improvement of the characteristics of defecation. Our findings point towards an anti-inflammatory mechanism of action of probiotics since most of the efficacy was shown for patients with a pro-inflammatory cytokine pattern in the duodenal fluid. This insight into the mechanism of probiotic action in IBS has never been shown before.

Available randomized studies are characterized by great controversy regarding the type of administered probiotics and the duration of treatment. A meta-analysis of 16 randomized trials concluded that most of benefit in the quality of life is coming from the administration of mixed preparations containing species of *Lactobacillus* and with treatment duration not longer than 4 weeks [18]. The significant decrease of the abdominal pain severity score among IBS-C and IBS-D irrespective of the presence of SIBO was a secondary study finding; this benefit being higher in patients with IBS-C. However, not all available studies showed a similar efficacy on abdominal pain severity, so far. In a randomized trial that enrolled 45 patients treated with *Saccharomyces boulardii* and 45 patients treated with placebo, an overall improvement of the quality of life was detected. Enrolled patients were suffering from IBS-D and IBS-M and they did not report any significant decrease of their abdominal pain [7]. A similar lack of effect on abdominal pain was reported in a randomized study of the administration of a mixture of seven probiotics compared to placebo among patients with IBS-D [9]. In another randomized trial where a mixture of *Bacillus subtilis* and *Streptococcus faecium* was administered to patients with IBS without diarrhea, a decrease of the abdominal pain score was reported [19].

In a recent randomized clinical trial, the efficacy of a liquid mixture of *Lactobacillus rhamnosus, Lactobacillus plantarum, Lactobacillus acidophilus*, and *Enterococcus faecium* in 124 patients was compared to that of placebo in 62 subjects. The administered preparation contained three species of *Lactobacillus*, exactly as it was the case with the probiotic mixture of our study. The achieved mean decrease of the overall symptom IBS score was 63.3% in the actively treated group compared to 28.3% of the placebo group [20]. This decrease was similar to the decrease reported for the IBS-SSS score among our IBS patients with SIBO*.* This last study [20] also evidenced a significant decrease of pain intensity, as it was the case with the study presented herein.

Although SIBO is most often present among patients with IBS-D [5, 6], our data show a beneficial effect of the intake of probiotics also for IBS-C patients, irrespective of the presence of SIBO. This positive effect may indicate that beyond SIBO treatment, probiotics may express their effect with a different mechanism of action. In a recent study, 34 IBS patients were randomized to treatment either with *Lactobacillus gasseri* or placebo for 4 weeks. Whole blood was collected at the end of treatment and at 4 weeks after the end of treatment for genomic expression. It was striking to identify a significant down-regulation of the EIF2 signaling pathway in the treatment arm that is associated with stress-related adverse behaviors [21].

Our study presents major strengths and several limitations. A major strength is the use of Rome III criteria of classification in the inclusion of patients. The limitations of the study are as follows: (a) the non-randomized design; (b) the small sample in the SIBO positive group; (c) the lack of analysis of the 16S rDNA gene; and (d) the lack of evaluation of SIBO using breath test. The largest randomized trials ever conducted for IBS are the TARGET I and TARGET II studies in which the efficacy of oral treatment with rifaximin was compared to placebo. The response rate of the placebo arm in the two TARGET studies did not exceed 32% [22]. As a consequence, we do believe that the goal of at least 50% decrease of IBS-SSS set in our study is of clinical significance. Furthermore, the achievement of a positive response rate in all patients with SIBO makes our findings of significance despite the small sample size. In our era, SIBO characterization should rely on sequencing of the 16S rDNA gene as already described in a recent study of our group. That study showed decrease of microbial diversity in the small intestine of patients with IBS so that species that predominate are also isolated with conventional microbiology [23]. This limits the impact of the lack of 16S rDNA sequencing in the interpretation of our findings. Glucose and lactulose breath tests are the gold standards for the diagnosis and follow-up of SIBO [1–4]. Their absence in our study design should be acknowledged as a limitation.

The studied probiotic preparation contained *Saccharomyces boulardii*. Although several concerns have been addressed on the safety of probiotics preparations enriched with yeasts, analysis have failed to disclose this [24]. However, it is empirically suggested that these preparations should be avoided in patients with severe immunodeficiency and neutropenia.

Our study prospectively demonstrated for the first time using very strict definition criteria that IBS patients with SIBO benefited most from the administration of this specific multi-strain preparation of probiotics. The study also demonstrated that many clinical aspects mainly related with improvement of stool form and satisfaction from bowel function after probiotics treatment are seen among patients either with IBS-D or IBS-C, irrespective of the presence of SIBO.
